# Calcification of extruded intervertebral discs in dachshunds: a radiographic, computed tomographic and histopathological study of 25 cases

**DOI:** 10.1186/s13028-019-0448-2

**Published:** 2019-03-08

**Authors:** Øyvind Stigen, Taizha Ciasca, Øyvor Kolbjørnsen

**Affiliations:** 1 Department of Companion Animal Clinical Sciences, Faculty of Veterinary Medicine, Norwegian University of Life Sciences, Sentrum, P.O. Box 369, 0102 Oslo, Norway; 2 Department of Pathology, National Veterinary Institute, Sentrum, P.O. Box 750, 0106 Oslo, Norway

**Keywords:** Calcification, Dachshund, Diagnostic imaging, Disc extrusion, Histopathology, Intervertebral disc disease

## Abstract

**Background:**

Three Nordic countries have national breeding programs to reduce the frequency of intervertebral disc disease in dachshunds. The programs include a radiographic examination of the vertebral column and dachshunds with more than four calcified discs visible on radiographs (CDVR) are discouraged from use in breeding. However, disc extrusion is also diagnosed in dachshunds without CDVR. The utility of the breeding programs is therefore questioned.

**Results:**

A prospective study of 25 dachshunds surgically treated for disc extrusion was conducted. For all the dogs, preoperative radiographs were evaluated for detectable disc calcifications and preoperative computed tomography (CT) scans were evaluated for presence of calcified material in the vertebral canal. Postoperatively, extruded disc material was examined for degeneration and calcification by histology. Diagnostic imaging and histology were done independently. Radiographically visible calcification was identified in 17 (68.0%) of 25 extruded discs. Calcification was seen in the disc space for all these 17 discs, and for eight of the 17, there was also calcified material visible in the vertebral canal. Extruded material from all the 25 discs was found to be calcified, both by CT and histopathology.

**Conclusions:**

In dachshunds with acute disc extrusion, radiographically visible calcification will frequently be found in the affected disc space, but not all affected disc spaces contain radiographically visible calcification. Using histopathology as the gold standard, a sensitivity of 0.3 (8/25) for radiography and 1.0 (25/25) for CT was found for detecting calcified disc material in the vertebral canal. Further, a sensitivity of 0.7 (17/25) was found for radiography for detecting remaining calcified material in the disc space. Thus, extruded disc material should be considered to be calcified, even in the absence of radiographically visible calcification.

## Background

Chondrodystrophic canine breeds are predisposed to degenerative changes in the intervertebral discs [1]. The degenerative changes have an early onset and include a chondroid metamorphosis with subsequent mineralization (calcification) of the nucleus pulposus. Degeneration and calcification significantly increase the risk for extrusion of the intervertebral disc, whereby nuclear material typically is displaced into the vertebral canal [1–3].

Dachshunds have the highest breed prevalence of disc extrusion and thereby also of intervertebral disc disease (IDD) [4–6]. In this breed, a positive correlation exists between the number of CDVR of young dogs, and the occurrence of IDD later in life [7–9]. A genetic factor is present for development of CDVR and with a heritability from 0.15 to 0.87 [10–12]. In Denmark, Finland and Norway, breeding programs have been initiated to reduce the frequency of CDVR in dachshunds, with the intention of lowering the occurrence of IDD.

In a retrospective study of 95 dachshunds surgically treated for disc extrusion, Rhodin et al. [13] found visible radiographic calcification in 54 (54.0%) of totally 100 extruded discs. Since calcification was detected only in just over half of the extruded discs, the value of a breeding program selecting dogs on the basis of CDVR was questioned. However, Rhodin et al. [13] included clinical cases in their study, whereas the ongoing breeding programs involve a radiographic screening of dachshunds without clinical signs. Consequently, the question arises whether the sensitivity of a radiographic examination to detect calcification in a disc is changed by extrusion.

If a calcified disc extrudes, mineralized material will leave the disc space and usually be spread out in the vertebral canal. Thus, lateral radiographs may show disc material at the intervertebral foramen above the disc, with the amount of calcified material remaining in the disc space being reduced. As a consequence, a change in radiographic sensitivity regarding calcified discs seems likely.

When extrusion of a disc without radiographically detectable calcification is diagnosed, it may either result from extrusion of non-calcified material or extrusion of calcified material that is not visible on radiographs. A histopathological examination of the extruded material can determine whether the material is calcified or not.

This study presents the frequency of CDVR in a material of 25 extruded discs diagnosed in Norwegian dachshunds. By using histopathology as the gold standard, the sensitivity of radiography and CT to detect calcification in extruded discs is determined.

## Methods

The study was designed as a prospective single-centre case series. The material included radiographs, CT-images and tissue samples of 25 extruded intervertebral discs in 25 dachshunds. The dogs were client-owned patients at the Faculty of Veterinary Medicine, Norwegian University of Life Sciences that showed progressive neurological deficits related to disc extrusion. Time from onset of clinical signs to removal of disc material from the vertebral canal ranged from 1 to 7 days in 17 dogs, 8 to 14 days in 4 dogs and 15 to 21 days in 4 dogs.

Eligible cases were recorded consecutively. Where plain radiography and CT were not sufficient to make the diagnosis of an extradural spinal cord compression, myelography, CT-myelography and magnetic resonance imaging (MRI) were available as additional diagnostic imaging modalities.

The case material was obtained over 5 years (2013–2017). The distribution of dachshunds into size and coat varieties, sex, age, neurological status and localization of disc extrusion is presented in Table 1. The mean age was 6.2 years (range 4–12 years) and 16 (64.0%) were males. Non-ambulatory paraparesis or paraplegia was found in 20 (80.0%) dogs, of which five also showed an absence of deep pain nociception.

**Table 1:** The case material of 25 dachshunds distributed by signalment, presurgical neurological status and localization of disc extrusion

Dog no.	Variety	Sex	Age, years	Neurological status, grade^a^	Extruded disc, localization
	Size, coat				
1	Kaninchen, smoothcoated	♂	6	2	L5–L6
2	Dwarf, smoothcoated	♂	7	3	T12–T13
3	Dwarf, wirehaired	♂	5	5	T12–T13
4	Dwarf, longhaired	♀	6	4	T13–L1
5	Dwarf, longhaired	♂ (castr.)	6	2	T12–T13
6	Dwarf, longhaired	♂	6	4	T11–T12
7	Dwarf, longhaired	♂	12	5	T11–T12
8	Standard, smoothcoated	♀	4	3	T11–T12
9	Standard, smoothcoated	♀	4	3	T12–T13
10	Standard, smoothcoated	♀	5	4	T11–T12
11	Standard, smoothcoated	♂	5	4	T13–L1
12	Standard, smoothcoated	♀	6	2	C2–C3
13	Standard, smoothcoated	♀	6	2	T9–T10
14	Standard, smoothcoated	♂	6	4	T13–L1
15	Standard, smoothcoated	♂	7	3	T10–T11
16	Standard, wirehaired	♂	4	3	T12–T13
17	Standard, wirehaired	♂	4	4	T12–T13
18	Standard, wirehaired	♀	5	4	T11–T12
19	Standard, wirehaired	♂	6	4	T13–L1
20	Standard, wirehaired	♂	7	5	T11–T12
21	Standard, wirehaired	♂	8	2	L1–L2
22	Standard, longhaired	♀	6	5	T11–T12
23	Standard, longhaired	♀	6	5	T13–L1
24	Standard, longhaired	♂	8	4	T11–T12
25	Standard, longhaired	♂	10	4	L2–L3

^a^Neurological status, grade 1: spinal pain without neurological deficits; 2: ambulatory paraparesis or tetraparesis and ataxia; 3: nonambulatory paraparesis or tetraparesis; 4: paraplegia or tetraplegia with intact deep pain nociception; 5: paraplegia or tetraplegia with absent deep pain nociception

The dogs were premedicated with 5–60 µg/kg medetomidine (Dorbene vet; Laboratorios SYVA SA) im or 0.01–0.03 mg/kg acepromazine (Plegicil vet; Pharmaxim AB) im and an iv or im injection of 0.1–0.3 mg/kg methadone hydrochloride (Metadon; NAF) for the radiographic examinations. Plain lateral radiographs were taken of the spines covering all segments in accordance with the neuroanatomic localization of the lesion. The radiographs were obtained with a Sound-Eklind DR system and viewed with Carestream PACs Workstations.

Anesthesia was induced by an iv injection of propofol (Norfol; Norbrook Laboratories Ltd), maintained with 1.6–2% isoflurane (IsoFlo vet; Zoetis) in oxygen and air delivered by means of a circle patient breathing system. CT of the spinal region of interest was performed with the dogs positioned in dorsal recumbency. The CT machine was a GE highspeed Fxi single slice helical scanner (GE Healthcare, Oslo Norway), the voltage was 120 kV and the milliampere–seconds used depended upon the size of the dog and varied between 140 and 160 mAs. The slice thickness was 2.5 mm and 1.25 mm in both soft tissue (WW 400; WL 40) and bone (WW 1900; WL 580) algorithms.

All radiographs and CT images were evaluated and reported by a board certified radiologist (Dipl ECVDI) and for all the 25 cases, probable disc material was identified in the vertebral canal and the extruded disc localized.

Following diagnostic imaging, 24 dogs proceeded directly to surgery whereas 1 dog was euthanased and submitted for post-mortem examination. In all dogs, the final diagnosis of a disc extrusion was confirmed and extruded disc material was sampled for histopathological examination.

A total of 53 specimens were collected from the 25 dogs. One single specimen was obtained from 12 dogs, while two to six pieces of extruded material were received from each of the remaining dogs. The largest specimen measured 14 × 4 × 4 mm, while the smallest measured 1 mm in diameter.

All specimens were fixed in 4% phosphate-buffered formaldehyde, pH 7.2. Subsequently the material was dehydrated in ethanol, equilibrated in xylene and embedded in paraffin. Decalcification of the specimens was not performed. Each specimen was sectioned at about 3 µm and stained with hematoxylin–eosin (HE) and von Kossa, a method for demonstration of calcium [14]. Blinded to the imaging results, one pathologist (ØK) evaluated all histological sections by light microscopy.

When all cases were obtained, two of the authors (ØS and TC) re-evaluated the radiographs independently and reported whether calcified disc material was visible in the vertebral canal or in the affected intervertebral disc space. The degree of calcification in the disc space was recorded for each dog. The decisions were made according to previously described semi-quantitative methods [15–17]. However, for two dogs the two authors recorded different degrees of calcification in the affected disc space. Radiographs of these two dogs were then re-evaluated by the two authors together, whereby a consensus was made. One board certified radiologist (TC) also re-evaluated the CT images and reported whether calcified material was present in the vertebral canal.

For one dog (no. 13), extra lateral radiographs of the thoracolumbar spine were available for comparison. The extra radiographs were taken at the NMBU-FVM 3 months before the dog presented with progressive neurological deficits as a result of disc extrusion at T9–T10. Upon the previous radiographic examination, the dog showed signs of temporary back pain only.

## Results

Upon radiographic examination, calcification was found in 17 (68.0%) of the 25 extruded discs. For all the 17 CDVR, calcified material was identified in the disc space whereby a severe degree of calcification was found in three, a moderate degree in six and a slight degree in eight spaces. For eight of the 17 CDVR, calcified material was also seen in the vertebral canal.

**Fig. 1:**
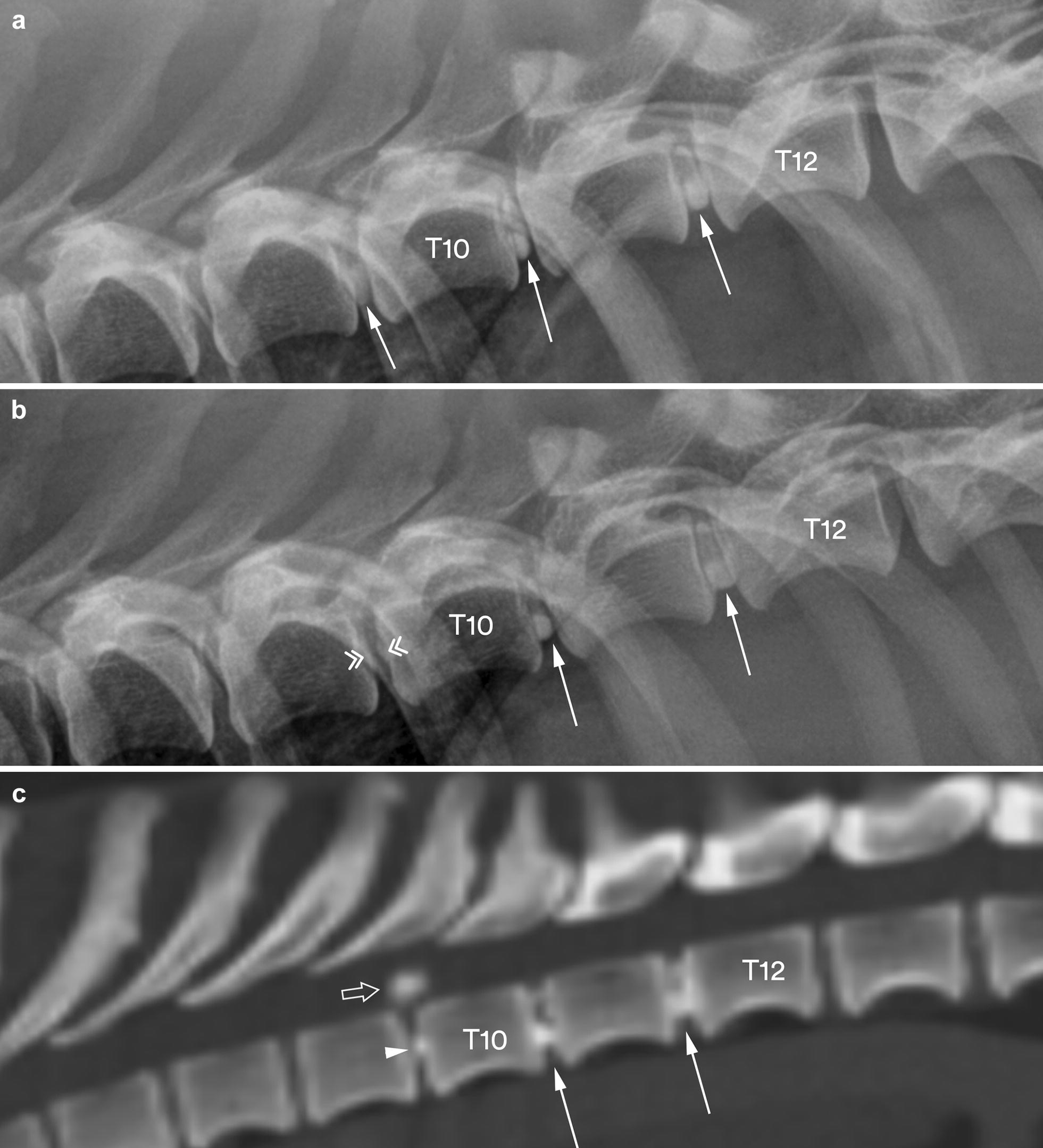
Lateral radiographs (**a**, **b**) and a sagittal multiplanar reconstruction (MPR) CT image in bone reconstruction algorithm (**c**) of the caudal thoracic vertebral column segment in dog no. 13. The radiographs are taken before (**a**) and after (**b**) the dog incurred a clinically significant disc extrusion. At the first presentation, three calcified intervertebral discs are identified (arrows) with a moderate degree of calcification in the T9–T10 disc space. At the second presentation, calcification is no longer visible in the T9–T10 disc space (double arrowheads). However, the concurrent CT image (**c**) shows calcified material in the vertebral canal (open arrow) above a slightly calcified T9–T10 disc space (arrowhead). The CT scan also confirms calcified material in the T10–T11 and T11–T12 disc spaces (solid arrows)

For dog no. 13, calcification was not radiographically visible in the disc space (T9–T10) after extrusion. However, on the extra lateral radiographs that were taken 3 months earlier, the same disc space (T9–T10) was found to have a moderate degree of calcification (Fig. 1).

**Fig. 2:**
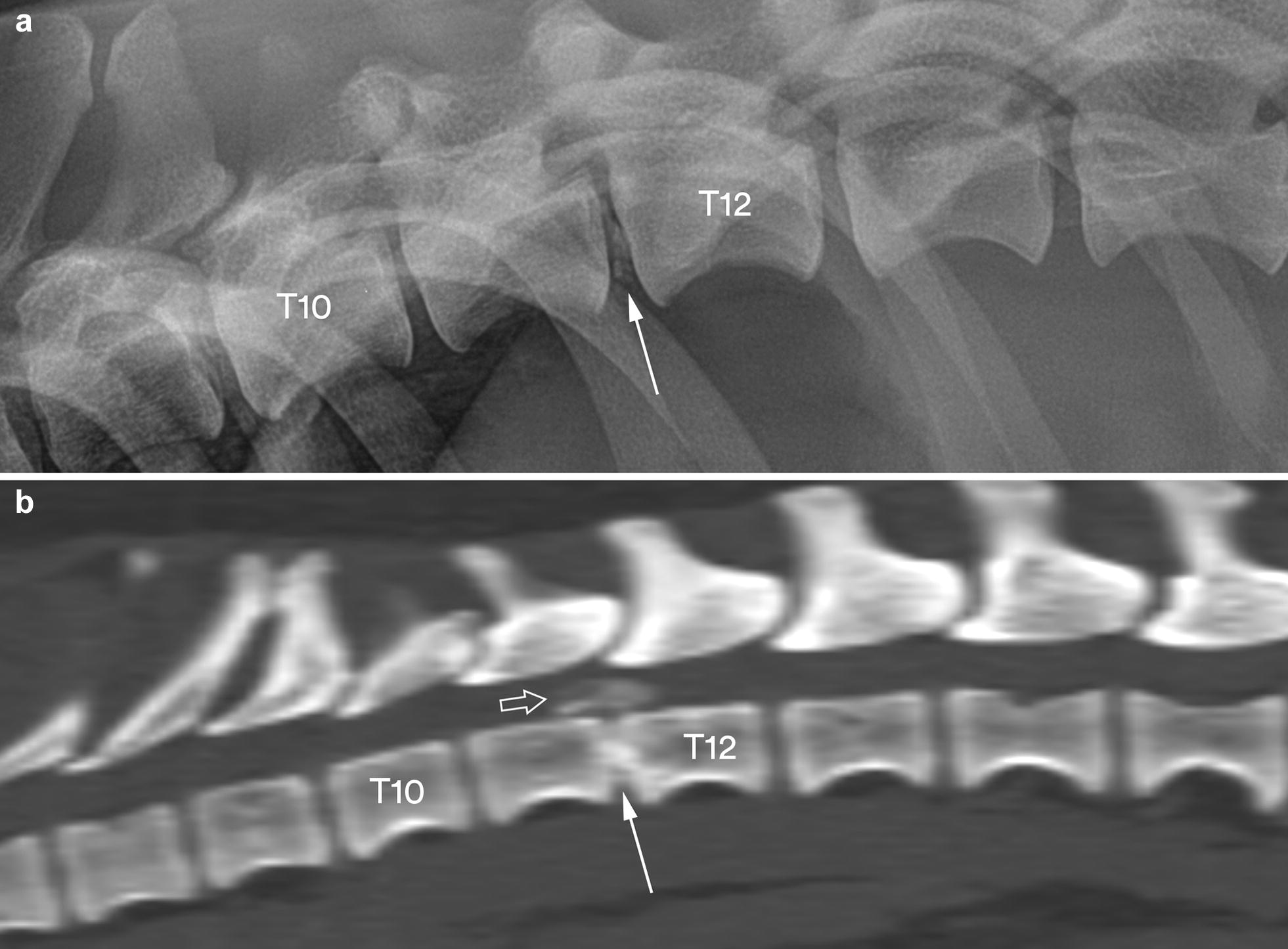
Lateral radiograph (**a**) and a sagittal MPR CT image in bone reconstruction algorithm (**b**) of the caudal thoracic and cranial lumbar vertebral column segments in dog no. 24. The radiograph shows a slight degree of calcification in the T11–T12 disc space (arrow), whereas the CT image reveals calcified material in the vertebral canal (open arrow) above a calcified T11–T12 disc space (solid arrow)

**Fig. 3:**
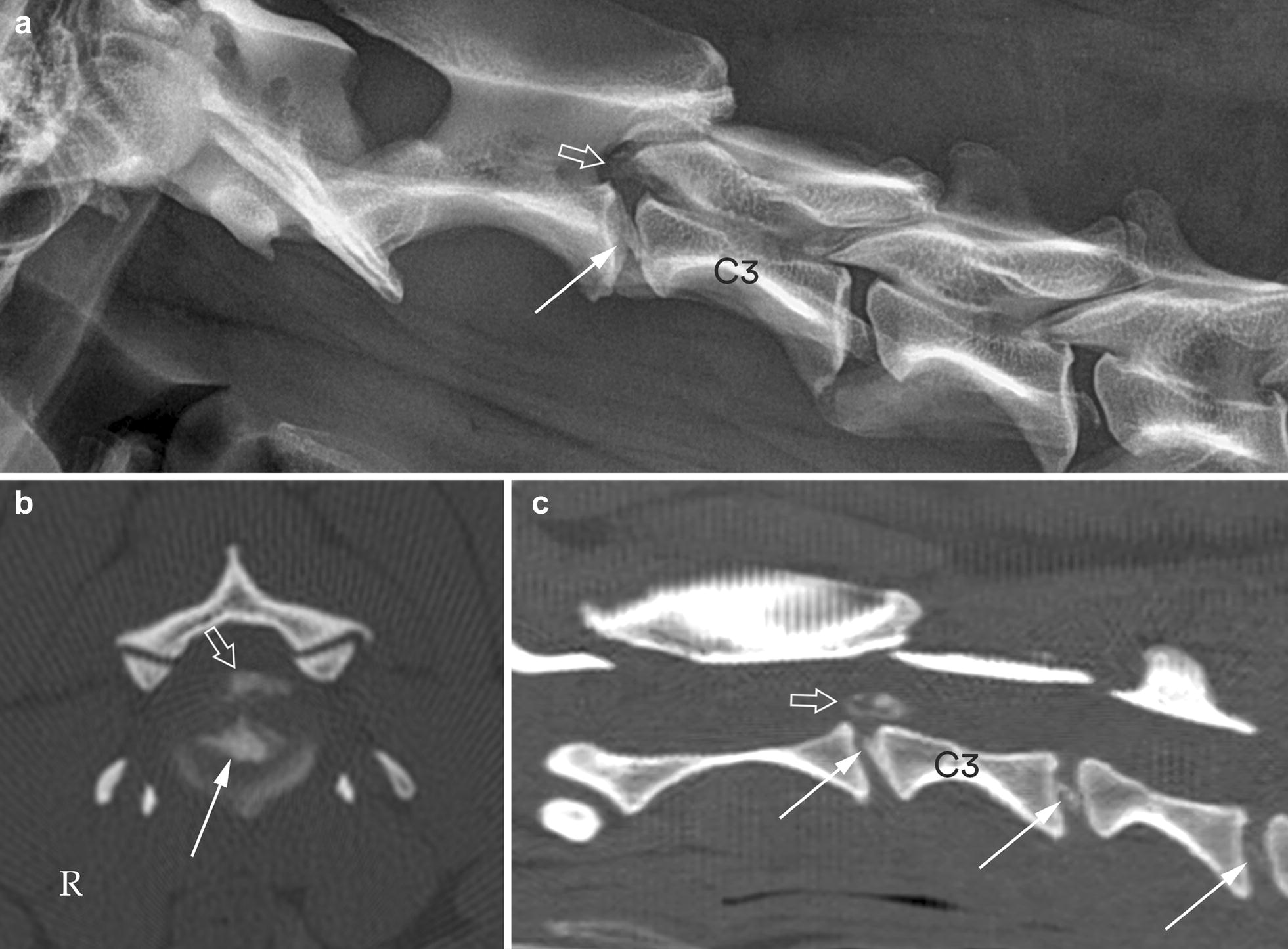
Lateral radiograph (**a**) and transverse and sagittal MPR CT images in bone reconstruction algorithm (**b**, **c**) of the cranial cervical vertebral column segment in dog no. 12. The radiograph shows calcified material in the vertebral canal (open arrow) above a moderately calcified C2–C3 disc space (solid arrow). The transverse CT image through the C2–C3 intervertebral disc (**b**) and the sagittal CT image (**c**), both reveal calcified material in the vertebral canal (open arrows) above a calcified C2–C3 intervertebral disc (solid arrows). The sagittal CT image also shows calcification in the C3–C4 and C4–C5 discs (solid arrows)

**Fig. 4:**
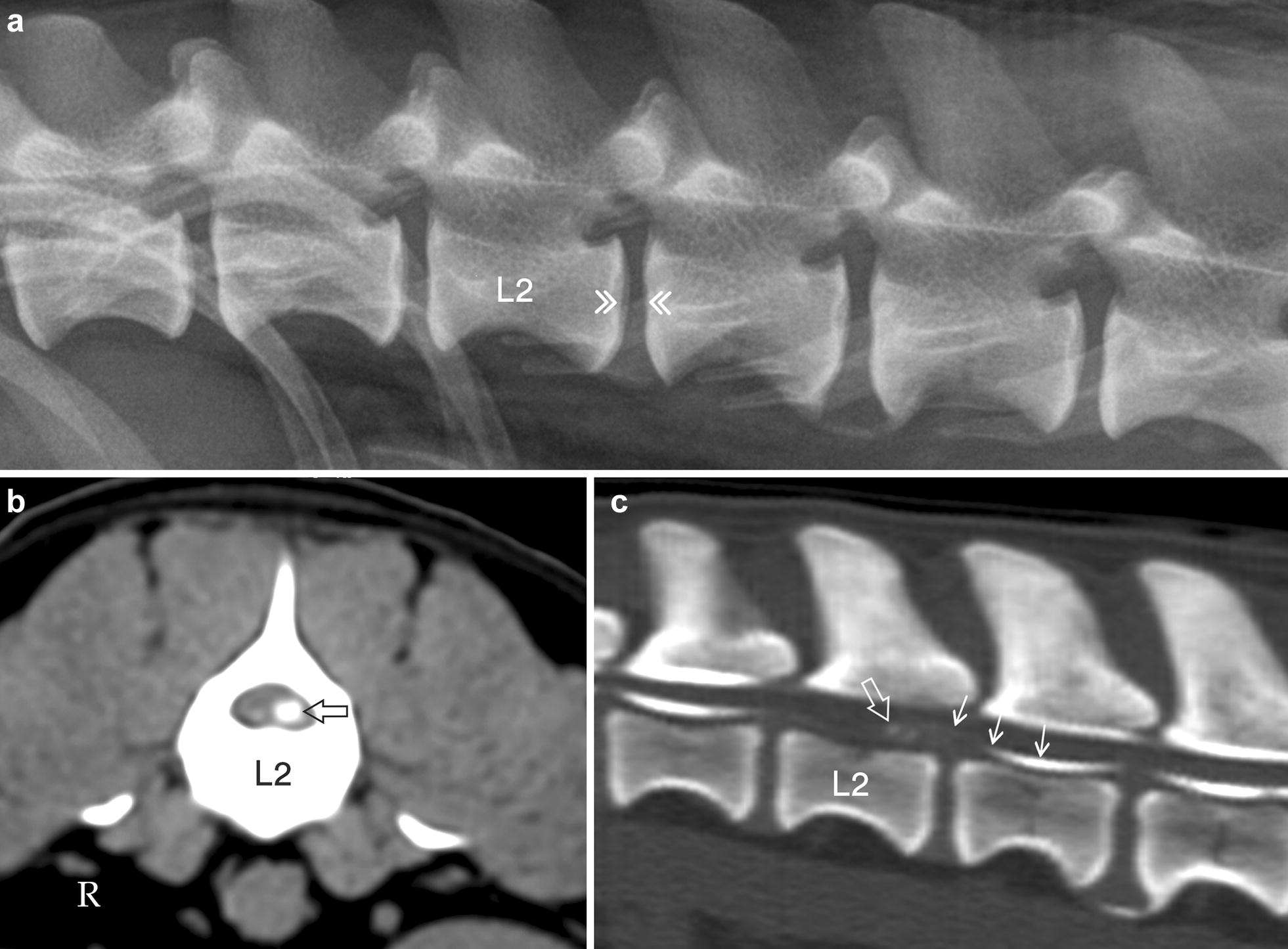
Lateral radiograph (**a**), transverse MPR CT image in soft tissue reconstruction algorithm (**b**) and sagittal MPR CT-myelogram in bone reconstruction algorithm (**c**) of the cranial lumbar vertebral column segment in dog no. 25. The radiograph reveals no calcification in the L2–L3 disc space (double arrow heads), whereas the CT image through the caudal part of L2 (**b**) shows calcified material on the ventral left side of the vertebral canal (open arrow). The CT-myelogram confirms the presence of calcified material in the vertebral canal (open arrow), but also demonstrates an extradural location of the lesion as the ventral subarachnoid contrast column is displaced dorsally (short arrows)

Calcified material was found in the vertebral canal of all the 25 dachshunds by CT. An extradural spinal cord compression was diagnosed in 24 (96.0%) of the dogs without using other modalities than plain radiography and CT (Figs. 2, 3). In the remaining dog (no. 25), a CT-myelogram was needed to determine the extradural location of the lesion (Fig. 4).

Histopathological examination revealed that degeneration and mineralization were present in extruded disc material from all (100.0%) the 25 dogs. For 21 dogs (84.0%), detection of neutrophil granulocytes showed that an active inflammation was also present.

The mineralized disc material identified by histopathology consisted of two different types: either a uniform basophilic granular material or irregular crystalline basophilic/purple fragments of various sizes. The calcification was verified by von Kossa staining in all specimens. Both types were seen in various amounts within the same sections in all intervertebral discs examined. However, in 22 discs (88.0%) there was a predominance of basophilic granular material.

**Fig. 5:**
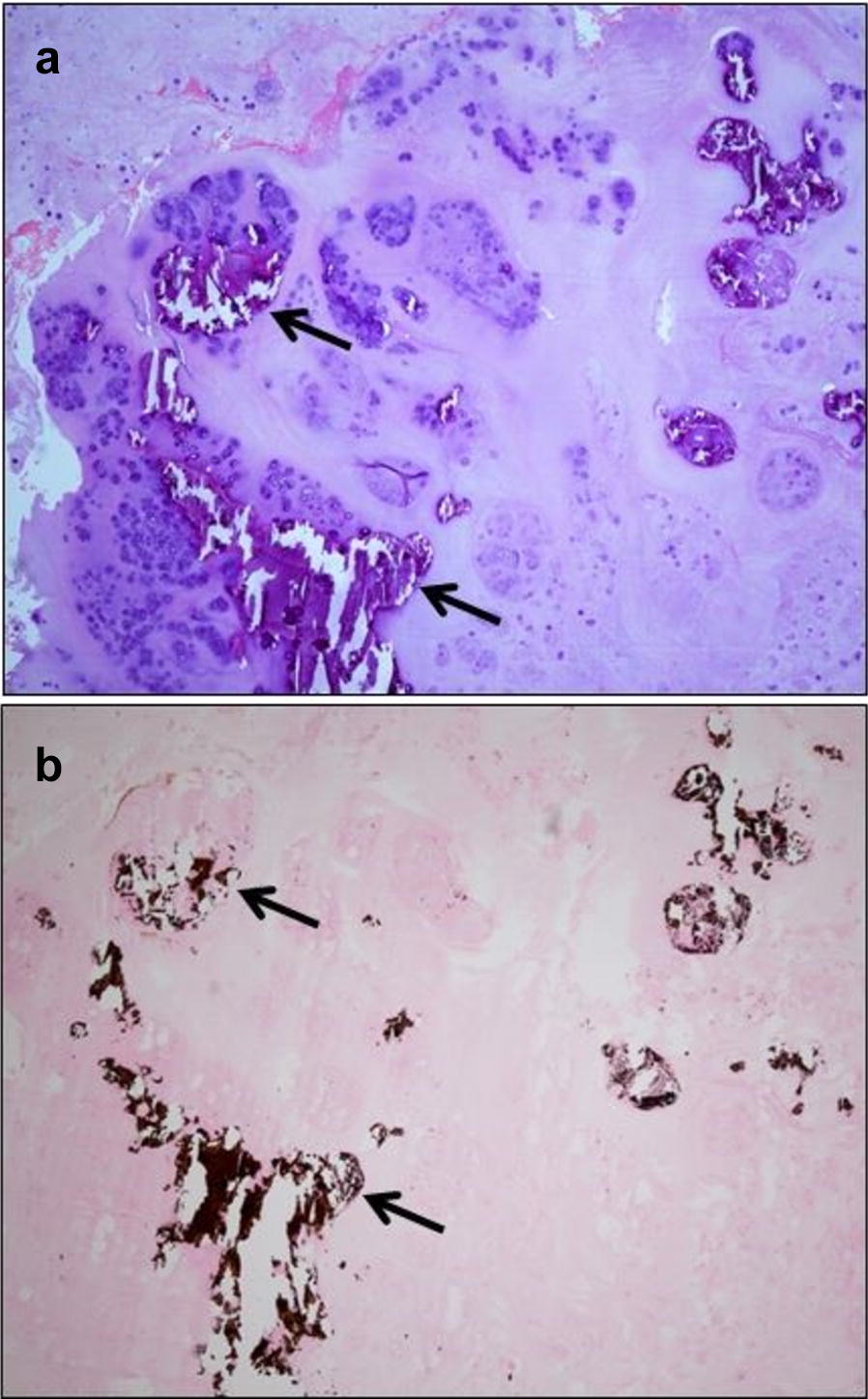
Photomicrographs of extruded disc material from the L2–L3 intervertebral disc in dog no. 25. In this dog, calcification was not detected on preoperative radiographs, neither in the L2–L3 disc space nor in the vertebral canal (see Fig. 4a). **a** Multiple crystalline fragments of various sizes (arrows) in extruded nucleus pulposus tissue. HE staining (Obj. ×40). **b** Calcification in the extruded nucleus pulposus, verified by brownish black deposits (arrows). Von Kossa staining (Obj. ×40)

Evidence of mineralization within the extruded disc material was also noted in the eight intervertebral discs without radiographic detectable calcification. In these eight discs, a predominance of basophilic granular material was found in five while irregular crystalline fragments were the main finding in the remaining three (Fig. 5).

By using histopathology as the gold standard, a sensitivity of 1.0 (25/25) was calculated for CT and of 0.3 (8/25) for plain radiography in identifying calcified material in the vertebral canal. Further, a sensitivity of 0.7 (17/25) was calculated for plain radiography in identifying remaining calcified material in the disc space.

## Discussion

Histopathology revealed calcification in extruded disc material from all dogs. To the authors’ knowledge, histologically confirmed extrusion of non-calcified material in dachshunds has not been reported. Thus; in dachshunds, extruded disc material should always be expected to have some degree of calcification.

By using histopathology as the gold standard, a significantly higher sensitivity (1.0 versus 0.3) was found for CT compared with radiography in identifying calcified material in the vertebral canal. The very high sensitivity of CT makes this modality a highly recommended imaging method for dachshunds with clinical signs of IDD.

Radiographic examination did not identify calcification in the disc space or in the vertebral canal of eight (32.0%) of the extruded discs. However, for one dog (no. 13) without radiographically visible calcification in the affected disc space (T9–T10) at the time of extrusion, a moderate degree of calcification had been found in the same disc space 3 months earlier. Pre-extrusion spinal radiographs were not available for any of the other 24 dachshunds. If pre-extrusion spinal radiographs had been available, it is reasonable to expect that calcification would have been observed in some of the other disc spaces that lacked radiographically visible calcification at the time of extrusion. It is also probable that more of the 17 disc spaces with radiographically visible calcification at the time of extrusion would have shown a more pronounced calcification at an earlier time point.

In a previous study of 20 dachshunds with a mean age of 5.3 years and euthanised for reasons unrelated to research, Stigen and Kolbjørnsen [17] found histopathologically detectable calcification in 230 (45.7%) of 503 non-extruded intervertebral discs. In the present study, histopathologically detectable calcification was found in material from all 25 extruded discs. Consequently, calcification was found more than twice as often in extruded disc material as in a comparable sample of non-extruded discs. Calcification subsequent to degeneration therefore also seems to be a significant predisposing factor for IDD in dachshunds.

Radiographically detectable calcification was found in 17 (68.0%) of the 25 extruded discs. This percentage is slightly larger than that found by Rohdin et al. [13], who identified radiographic calcification in 54 (54.0%) of 100 extruded discs. The larger percentage found in the present study may be explained by a lower number of cases, a possible higher sensitivity at the radiographic examination or a true difference between the Norwegian and Finnish dachshund populations. Collectively, the two studies show that radiographic calcification is found in 71 (56.8%) of 125 extruded discs in Nordic dachshunds.

Only lateral views were included at the radiographic examination. By omitting ventrodorsal and oblique views, some radiographically detectable calcifications of extruded discs may have been overlooked. As a result, the recorded number of 17 (68.0%) extruded discs with radiographically visible calcification may be lower than the true number. However, lateral radiographs imply that any mineralized material at the disc spaces or at the intervertebral foramina within the vertebral canal will not be superimposed on vertebral bone and thereby better visualized. Thus; additional views were considered not to increase the sensitivity for detecting CDVR significantly. For the same reason, only lateral radiographs are required at the radiographic screening programs for CDVR in Danish, Finnish and Norwegian dachshunds. Rohdin et al. [13] made four lateral and four ventrodorsal radiographs of the vertebral column of each of the 95 dachshunds they examinated for CDVR, but no advantage from including the ventrodorsal views was reported.

The CT examination was found to have a sensitivity of 1.0 while a specificity was not calculated as histopathological calcification was revealed in extruded material from all the 25 dogs. CT imaging has the advantage of being extremely sensitive to changes in radiographic density [18, 19]. This modality is therefore suitable for visualizing even tiny amounts of calcified disc material in the vertebral canal, which the calculated sensitivity of 1.0 also shows.

MRI was available but not considered necessary to make the diagnosis of an extradural mass in any of the 25 dachshunds. Diagnosis was possible because there was calcification of the extruded material in all dogs combined with the very high sensitivity of CT to detect mineralized material. MRI is unrivaled in showing detail of spinal cord parenchyma and is usually the preferred modality to identify the site and size of vertebral canal masses in dogs [13, 20, 21]. However, when it comes to calcified masses, CT is considered to be more sensitive than MRI [19, 22, 23]. Thus, CT is found suitable as a first-line imaging modality for dogs, and especially for dachshunds, presenting with thoracolumbar myelopathy [24].

Histopathological studies of extruded disc material from dogs have been published previously [25–29], but to the authors’ knowledge, only one study [25] concerns specimens obtained exclusively from dachshunds. However, in that study inflammation was well described while information about mineralization is not included. In other histopathological studies [26–29], the percentage of dachshunds from which disc material is collected, ranges from 9.1 [29] to 51.1 [26]. In the study with the highest percentage [26], specimens were obtained intraoperatively from a total of 45 dogs, of which 23 (51.1%) were dachshunds. Variable amounts of granular, basophilic, mineralized cartilage were detected in specimens from 34 dogs, but the number of dachshunds among these is not reported. All specimens were stained with HE, but not with von Kossa. Thus; as previous studies and our present study are based on different protocols, their results can not be directly compared.

In this study, two patterns of mineralization were identified by light microscopy; one comprised basophilic granular material, while the other showed crystalline fragments of various size. Crystallographic studies to determine the precise composition of the calcific deposits were not performed. However, further biochemical studies of the nature of calcified material in intervertebral discs in dachshunds are of interest. The composition could possibly explain the negative radiographs.

## Conclusions

In dachshunds, extruded disc material should be considered calcified and therefore detectable with CT. However, upon radiographic examination, calcified disc material in the vertebral canal is often undetectable.

In dachshunds with IDD, calcified material in the vertebral canal may originate from intervertebral disc spaces with or without radiographically visible calcification.

## Abbreviations

CDVR: calcified discs visible on radiographs
CT: computed tomography
IDD: intervertebral disc disease
MRI: magnetic resonance imaging
